# The impact of ginsenosides on cognitive deficits in experimental animal studies of Alzheimer’s disease: a systematic review

**DOI:** 10.1186/s12906-015-0894-y

**Published:** 2015-10-24

**Authors:** Chenxia Sheng, Weijun Peng, Zi-an Xia, Yang Wang, Zeqi Chen, Nanxiang Su, Zhe Wang

**Affiliations:** Department of Integrated Chinese and Western Medicine, The Second Xiangya Hospital, Central South University, No.139 Middle Renmin Road, Changsha, Hunan 410011 PR China; Institute of Integrative Medicine, Xiangya Hospital, Central South University, Changsha, 410008 Hunan PR China; Key Laboratory of Chinese Gan of State Administration of Traditional Chinese Medicine of China, Changsha, 410008 Hunan PR China

## Abstract

**Background:**

The efficacy of ginsenoside treatment on cognitive decline in individuals with Alzheimer’s disease (AD) has yet to be investigated. In this protocal, we conducted a systematic review to evaluate the effect of ginsenosides on cognitive deficits in experimental rodent AD models.

**Methods:**

We identified eligible studies by searching seven electronic databases spanning from January 1980 to October 2014. We assessed the study quality, evaluated the efficacy of ginsenoside treatment, and performed a stratified meta-analysis and meta-regression analysis to assess the influence of the study design on ginsenoside efficacy.

**Results:**

Twelve studies fulfilled our inclusion criteria from a total of 283 publications. The overall methodological quality of these studies was poor. The meta-analysis revealed that ginsenosides have a statistically significant positive effect on cognitive performance in experimental AD models. The stratified analysis revealed that ginsenoside Rg1 had the greatest effect on acquisition and retention memory in AD models. The effect size was significantly higher for both acquisition and retention memory in studies that used female animals compared with male animals.

**Conclusions:**

We conclude that ginsenosides might reduce cognitive deficits in AD models. However, additional well-designed and well-reported animal studies are needed to inform further clinical investigations.

## Background

Alzheimer’s disease (AD), the incidence of which is rapidly increasing worldwide, leads to death within 3 to 9 years after diagnosis. It has been estimated that the number of individuals living to 100 years or more will increase by over 200 % and that the number of individuals surviving to 90–95 years will double between 2000 and 2020 [1]. One of the most significant disabilities associated with AD is cognitive impairment [2]. This impairment interferes with work, relationships, leisure, and activities of daily living and exacts a personal and economic cost that is difficult to quantify. In addition, cognitive impairment in AD is associated with a significant social burden and time commitment on the caregiver [3].

AD is characterized by three neuropathological hallmarks: neuronal loss, senile plaques (SPs), and neurofibrillary tangles (NFTs) [4, 5]. SPs predominantly consist of extracellular amyloid β peptide (Aβ), which is the key protein that induces neuronal damage and apoptosis in AD patients [6, 7]. NFTs are formed by intraneuronal aggregation of hyperphosphorylated tau [8]. Few therapeutic options to prevent or alleviate cognitive deficits in AD exist [9].

Ginseng, the root of Panax ginseng C.A. Meyer, is a traditional medicinal herb that has been applied widely in Asia for the treatment of aging and memory deterioration [10]. Ginsenosides such as ginsenoside Rg, ginsenoside Rb, and ginsenoside Re are the most important pharmacologically active ingredients in ginseng [11]. Ginsenosides are a class of tetracyclic triterpene glycosides (also known as saponins) [12] that are widely used in herbal medicine and have been shown to attenuate cognitive impairment and improve behavioral symptoms in humans [13]. Recent studies have demonstrated that the long-term consumption of ginsenoside Rg1 improves cognitive performance, decreases the levels of Aβ_1–42_ protein in the hippocampus of aged senescence-accelerated mouse prone 8 (SAMP8) [14], decreases the accumulation of NFTs [15], increases the extracellular secretion of soluble amyloid precursor protein α (sAPPα), and enhances α-secretase activity [16]. Rb1 protects neurons against Aβ_1–42_-induced neurotoxicity and tau hyperphosphorylation [17]. Furthermore, ginsenoside Rh2 reduces senile plaques and plays an important role in neuronal differentiation [18]. Ginsenoside Rd has been shown to alleviate inflammation and alleviate cognitive deficits [19].

The need to conduct systematic reviews of animal experiments, which can provide evidence for the potential translational value of animal models to human disease, has been highlighted [20]. Systematic reviews and meta-analyses of animal studies contribute to the modeling of clinically relevant problems; in particular, such reviews allow decisions regarding the design and conduct of subsequent clinical trials to be based on all existing evidence synthesized in an unbiased manner. Moreover, systematic reviews permit a more objective appraisal of evidence than is allowed by the traditional narrative-style reviews that are more commonly associated with animal research [21]. However, there are few systematic randomized trials and observational studies that have explored the effect of ginsenosides on cognition in humans with AD. Therefore, a meta-analysis was conducted to evaluate the efficacy of ginsenosides for the treatment of cognitive impairment in experimental animal models of AD and explore the impact of the study design and quality on the reported outcomes.

## Methods

### Literature search

In October 2014, seven electronic databases (PubMed, Web of Science, MEDLINE, Embase, Google Scholar, CNKI, and Wanfang) were searched using the terms “Alzheimer’s disease” (or “dementia”, “Alzheimer disease”, “Alzheimer”, “Alzheimer’s”, “Alzheimers”) and “ginsenoside” (or “ginseng, “ginseng saponin”). All the searches were limited to literature published between January 1980 and October 2014. This systematic review was limited to the results of animal studies. The reference lists from relevant publications were used to identify further relevant research articles and reviews. Each study had to meet several inclusion criteria (Table 1) to be included in this meta-analysis. To determine their eligibility for inclusion, two investigators (Chenxia Sheng and Weijun Peng) assessed the titles and abstracts of identified articles, and obtained copies of the articles to review the study design and methodology for studies that administered ginsenoside and measured cognitive, behavioral, and motor problems in AD model rodents. Disagreements among the investigators were resolved by consensus following a discussion.

**Table 1:** Criteria for study inclusion/exclusion

Inclusion criteria	Exclusion criteria
(1) Ginsenoside were administered.	(1) Ginsenoside were not administered.
(2) Experimental AD was induced in rodents (i.e., rats or mice).	(2) Other types of animals (e.g., sheep, cats, and dogs) were used.
(3) AD treatment group was treated with a pharmacological agent, and a control group was administered a placebo after injury.	(3) Treatment group was administered another neuroprotective agent in addition to ginsenoside.
(4) Cognitive function was measured by the MWM.	(4) Treatment group was administered another Chinese Traditional medicine in addition to ginsenoside.
(5) Article was published in English or Chinese language.	(5) Only biochemical or physiological outcomes of treatment efficacy were assessed.
	(6) No control group was used. (8) Duplicate publications.

### Data extraction

Two investigators extracted information about the studies, including the species; sample size; type of AD model; main experimental groups; type of anesthetic agent; substances used as experimental and control treatments; the dose, method, and timing of ginsenoside administration; and the time of the outcome assessment.

The Morris water maze (MWM) was used to assess cognition in all the studies included in the analysis. If cognition was assessed several times in the study, then only the final assessment was included in the analysis. For cases in which the data were expressed graphically, the investigators attempted to obtain numerical values from the study authors; if these values were not available, then digital ruler software was used to estimate the numerical values from the graphs. For cases in which the data were missing, the investigators contacted the authors and requested the additional information. If the required data were not available, then the study was excluded from the analysis. If one study examined different animal models of AD or ginsenoside doses, then these models or doses were analyzed as separate studies.

### Methodological quality

The methodological quality of the studies was assessed based on a checklist modified from the Collaborative Approach to Meta-Analysis and Review of Animal Data from Experimental Studies (CAMARADES), as previously described [22] with minor modifications. The modified CAMARADES included randomization of subjects into treatment groups instead of blinded induction of ischemia (allocation concealment) [23]. One point was tallied for written evidence of each of the following criteria: peer-reviewed publication; randomization of subjects into treatment groups; assessment of dose–response relationship; blind assessment of behavioral outcomes; monitoring of physiological parameters, such as body temperature; calculation of the sample size necessary to achieve sufficient power; statement of compliance with animal welfare regulations; avoidance of anesthetic agents with marked intrinsic neuroprotective properties (e.g., ketamine); statement of potential conflicts of interest; and use of a suitable animal model (Table 2).

**Table 2:** Characteristics of included studies

Study	Animal species	AD model	Main experimental groups	Method of administration	Time of ginsenoside administration	Anesthetic agent	Duration of supplementation
Wang YC et al. 2014	Male Kunming mice	chronic restraint stress (CRS)	Control r(*n* = 15)	Ig	Immediately after injury	chloral hydrate	8 weeks
			CRS + distilled water (*n* = 15)				
			CRS +2.0 mg/kg Rg1 (*n* = 15)				
			CRS +5.0 mg Rg1 /kg r(*n* = 15)				
Bombi Lee et al. 2013	Male SD rats	LPS injected into the bilateral lateral cerebral ventricle	LPS-injected plus saline (*n* = 6)	Ip	Immediately after injury	pentobarbital	3 weeks
			LPS-injected plus 10 mg/kg Rg3 (*n* = 6)				
			LPS-injected plus 20 mg/kg Rg3 (*n* = 6)				
			LPS-injected plus 30 mg/kg Rg3 (*n* = 6)				
Song XY et al. 2013	Male SD rats	OKA injected into the right lateral cerebral ventricle	OKA-injected plus distilled water (*n* = 12)	Ig	A week before OKA microinjected	chloral hydrate	25 days
			OKA-injected plus 5 mg/kg Rg1 (*n* = 12)				
			OKA-injected plus 10 mg/kg Rg1 (*n* = 12)				
			OKA-injected plus 20 mg/kg Rg1 (*n* = 12)				
Quan QK et al. 2013	Male SD rats	Aβ1–42 injected into Both Hippocampal CA1 regions	Aβ1–42 injected plus normal saline (*n* = 10)	Ip	5 days after Aβ1–42 injected	chloral hydrate	4 weeks
			Aβ1–42 injected plus 10 mg/kg Rg1 (*n* = 10)				
Zhao HH et al. 2012	Female ICR mice	Oral AlCl3 in drinking water	Al exposure plus distilled water (*n* = 8)	Ig	6 month after AlCl3 oraled	pentobarbital	4 months
			Al exposure plus 20 mg/kg Rb1 (*n* = 8)				
Wang YL et al. 2011	Male Wistar rats	Aβ1-40 into the right lateral cerebral ventricle	Aβ1-42 plus saline (*n* = 10)	Ip	2 weeks after AlCl3 orale	not clear	4 weeks
			A1-42 plus 10 mg/kg Rg2 (*n* = 10)				
Zhang X et al. 2012	Female Wistar rats	Ovariectomized (OVX) & D-gal injected intraperitoneally	OVX, D-gal plus vehicle	Ip	Immediately after injury	chloral hydrate	6 weeks
			OVX, D-gal plus 5 mg/kg Rg1 (*n* = 8)				
			OVX, D-gal plus 10 mg/kg Rg1 (*n* = 8)				
			OVX, D-gal plus 20 mg/kg Rg1 (*n* = 8)				
Chu SH et al. 2014	Wistar rats	Streptozotocin (STZ) injected into the bilateral lateral cerebral ventricle	STZ plus physiological saline (*n* = 12)	Ig	2 days after STZ injected	not clear	4 weeks
			STZ plus 5 mg/kg Rg5 (*n* = 12)				
			STZ plus 10 mg/kg Rg5 (*n* = 12)				
			STZ plus 20 mg/kg Rg5 (*n* = 12)				
Zhou LP et al. 2011	Female C57BL/6 mice	Ovariectomized (OVX) & Aβ_25-35_ injected into the lateral cerebral ventricle	OVX, Aβ 25–35 plus Rg1 10 mg/kg (*n* = 10)	Ip	10 days later	chloral hydrate	14 days
Wang XY et al. 2001	Male Kunming mice	Aβ_25–35_ injected into the lateral cerebral ventricle	Aβ25-35 plus physiological saline (*n* = 10)	Ip	1 days after Aβ25-35 injected	diethyl ether	10 days
			Aβ25-35 plus 5 mg/kg Rg5 (*n* = 10)				
			Aβ25-35 plus 10 mg/kg Rg5 (*n* = 10)				
Wu W et al. 2011	Male SD rats	fimbria/fornix transection	fimbria/fornix transection(15)	Ip	14 days after fimbria/fornix transection	chloral hydrate	4 weeks
			fimbria/fornix transection plus 10 mg/kg Rg1 (*n* = 15)				
Zang Y et al. 2010	Male & female Wistar rats	Aβ_25–35_ injected into Both Hippocampal regions	Aβ25-35 plus physiological saline (*n* = 12)	Ip	15 days before Aβ25-35 injected	chloral hydrate	5 weeks
			Aβ25-35 plus 3 mg/kg Rg2 (*n* = 12)				
			Aβ25-35 plus 6 mg/kg Rg2 (*n* = 12)				
			Aβ25-35 plus 12 mg/kg Rg2 (*n* = 12)				

*Ig* intragastrically, *Ip* intraperitoneally

The study quality also was assessed with secondary criteria as previously described [24] (Table 3). These criteria included study characteristics such as the age, species, and sex of the animals used; the duration of supplementation; and the dose(s) of ginsenoside. These criteria also included an assessment of the internal validity of the study, i.e., performance bias (differences in care provided?); exclusion bias (differences in withdrawal from studies?); detection bias (differences in outcome measurements?); and selection bias (differences in allocation to comparison groups?), as well as an assessment of the external validity of the population, intervention, and outcome.

**Table 3:** The CAMARADES quality items

Study	➀	➁	➂	➃	➄	➅	➆	➇	➈	➉	Quality score
Wang YC et al. 2014	√	√	√		√		√	√		√	7
Bombi Lee et al. 2013	√	√	√	√	√		√	√		√	8
Song XY et al. 2013	√	√	√				√	√		√	6
Quan QK et al. 2013	√	√					√	√	√	√	6
Zhao HH et al. 2012	√	√					√	√	√	√	6
Wang YL et al. 2010	√	√			√			?		√	4
Zhang X et al. 2012	√	√	√		√		√	√	√	√	8
Chu SH et al. 2014	√		√				√	?		√	4
Zhou LP et al. 2011	√	√						√		√	4
Wang XY et al. 2001	√	√	√				√	√		√	6
Wu W et al. 2011	√	√						√		√	4
Zang Y et al. 2010	√	√	√					√		√	5

(1) peer reviewed publication; (2) presence of randomization of subjects into treatment groups; (3) assessment of dose–response relationship; (4) blinded assessment of behavioural outcome; (5) monitoring of physiological parameters such as body temperature; (6) calculation of necessary sample size to achieve sufficient power; (7) statement of compliance with animal welfare regulations; (8) avoidance of anaesthetic agents with marked intrinsic neuroprotective properties (e.g., ketamine); (9) statement of potential conflict of interests; (10) use of a suitable animal model

### Statistical analysis

All statistical analyses were conducted in accordance with the *Cochrane Handbook for Systematic Reviews of Interventions*. For each outcome measure in each study, the standardized mean difference (SMD; equal to the difference in the mean outcome between the groups divided by the standard deviation of the outcomes among the participants, which was reported in units of standard deviation) was calculated, which allows data measured on different scales to be merged. Despite the anticipated heterogeneity, the individual SMDs were pooled whenever possible to obtain an overall SMD and 95 % confidence intervals (CI).

Within- and between-study heterogeneity or variation was assessed using Cochran’s Q-statistic. A significant Q-statistic (i.e., *p* < 0.10) indicated heterogeneity among studies. Heterogeneity also was assessed using the metric of Cochran’s Q-statistic, I^2^ values of 25, 50 and 75 % correspond to low, medium and high levels of heterogeneity, respectively; values ≤ 50 % indicated an acceptable degree of heterogeneity between studies [25].

The presence of small-study effects was investigated with funnel plots and Egger tests. A *p* < 0.10 on the Egger test indicated the presence of small-study effects. For studies comparing different doses or timing of drug administration to a single control group, the data from all experimental groups were pooled to compare with the control group. The pooled effect size was estimated using fixed- and random-effects models. When there was heterogeneity among studies, the pooled effect size was estimated using a random-effects model.

Both biological and methodological characteristics were examined in an attempt to explain the possible causes of heterogeneity (i.e., the possible causes modifying the outcome) among the studies. Stratified analyses were performed with experiments grouped according to the following characteristics: species and sex of the animals; anesthetic method; type and dose of ginsenoside; study quality; and the route of drug delivery. The difference between the groups was assessed by partitioning the heterogeneity and using the *X*^*2*^ distribution with *n-1* degrees of freedom (df), where *n* equals the number of groups. To adjust the significance levels for multiple comparisons, we used a Bonferroni correction [26] [declared significance = 1 − (1 − denoted significance)^(1/number of comparisons)], yielding critical p-values of 0.00215 for the acquisition memory and 0.00394 for the retention memory.

Finally, the impact of several variables (i.e., species and sex of the animals; anesthetic method; type and dose of ginsenoside; study quality; and the route of drug delivery) on the efficacy of ginsenoside was assessed using meta-regression when substantial or considerable heterogeneity existed.

All statistical analyses were performed using Stata software (version 13.0, College Station, Texas, USA) and Review Manager (version 5.3).

## Results

### Study inclusion

**Fig. 1:**
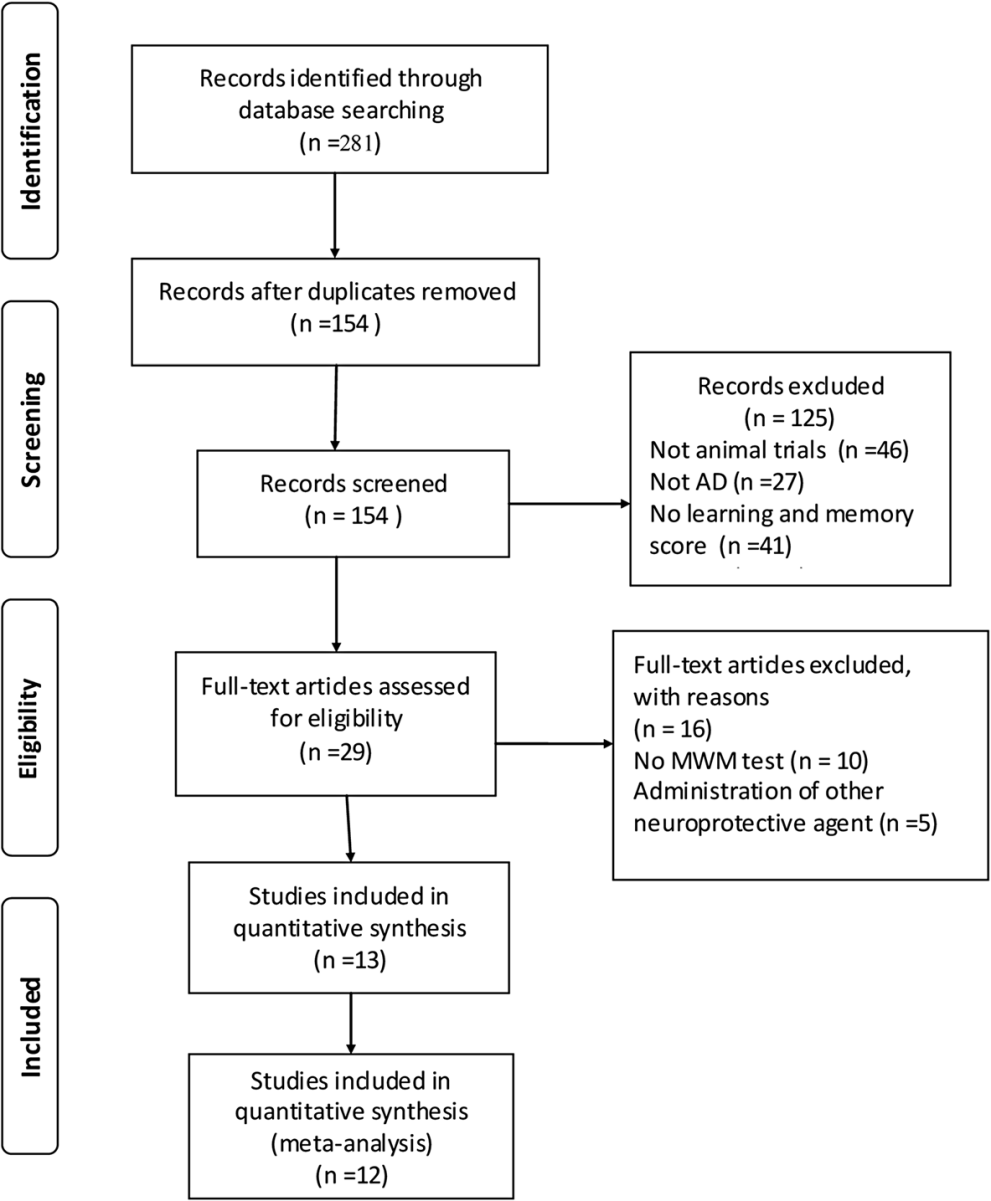
Flow diagram of study search process

A total of 281 publications were identified, of which 13 met our inclusion criteria [18, 27–38]. Of these 13 publications, one [18] was excluded from further analysis because too few data were available to assess in the meta-analysis. Therefore, this meta-analysis is based on 12 studies, which included 24 comparisons of acquisition memory and 13 comparisons of retention memory (Fig. 1).

### Study characteristics

Of the 12 included studies (Table 4), four were published in Chinese academic journals and 8 were published in English academic journals. The Aβ-induced AD model was the most frequently used animal model of AD [30–33, 35, 37]. Of the 12 studies, four used non-transgenic mice [27, 31, 35, 37], 4 used Sprague–Dawley rats [28–30, 36], and four used Wistar rats [32–34, 38]. Six studies used male animals only [27–30, 32, 36], and three studies used female animals only [31, 33, 35]. One study [38] used equal numbers of male and female animals. Two studies [34, 37] did not report the sex of the animals used. Across all studies, ginsenoside Rg1, ginsenoside Rg2, ginsenoside Rg3, ginsenoside Rg5, and ginsenoside Rb1 were administered as experimental treatments in doses of 1, 2, 3, 4, 5, 6, 9, 10, 20, or 30 mg/kg/day via either oral gavage or intraperitoneal injection. All studies used the MWM to assess cognitive function.

**Table 4:** Quality assessment of the included studies

Study quality:	Wang YC	Bombi Lee	Song XY	Quan QK	Zhao HH	Zhang X	Wang YL	Chu SH	Zhou LP	Wang XY	Wu W	ZangY
Research question specified and clear?	√	√	√	√	√	√	√	√	√	√	√	√
Outcome measures relevant for AD research	√	√	√	√	√	√	√	√	√	√	√	√
Are the characteristics of study population clear?												
Species	√	√	√	√	√	√	√	√	√	√	√	√
Background/generation	√	√	√	√	√	√	√	√	√	√	√	√
Sex (and distribution)	√	√	√	√	√	N	√	N	√	√	√	N
Age	N	√	√	√	√	√	√	√	√	√	√	√
Presence and correct control group?	√	√	√	√	√	√	√	√	√	√	√	√
Where the groups similar at baseline (if not randomized think of weight and sex etc.)?	√	√	√	√	√	N	√	N	√	?	√	N
Is the experiment randomized?	√	√	√	√	√	N	√	N	√	√	√	√
Kind of supplement mentioned (ginsenoside)?	√	√	√	√	√	√	√	√	√	√	√	√
Age when supplementation started mentioned?	√	√	√	√	√	√	√	√	√	√	√	√
Duration of supplementation clear and specified?	√	√	√	√	√	√	√	√	√	√	√	√
Amount of ginsenoside mentioned	√	√	√	√	√	√	√	√	√	√	√	√
Administration route specified	√	√	√	√	√	√	√	√	√	√	√	√
Is the timing of the supplementation during the day specified and similar in both groups?	√	√	√	√	√	√	√	√	√	√	√	√
Methods used for outcome assessment the same in both groups?	√	√	√	√	√	√	√	√	√	√	√	√
Did report animals who died or were otherwise removed from the study	N	N	N	N	N	N	N	N	N	N	N	N
Blinded outcome assessment?	N	√	N	N	N	N	N	N	N	N	N	N
Was the outcome assessment randomized across the groups?	N	N	N	N	N	N	N	N	N	N	N	N
Total number of animals included in statistical analyses clear?	√	√	√	√	√	√	√	√	√	√	√	√
Age of sacrificing animals mentioned?	√	√	√	√	√	√	√	√	√	√	√	√
Quality score (items√)	17	19	18	18	18	15	18	15	18	17	18	18

√ = fulfilling the criterion, no = not fulfilling the criterion, ? = not enough information to determine whether or not the raised criterion is

### Methodological quality of studies

A large variety of tools to assess the quality of animal studies is currently used, but none of these tools focus on internal validity only. Most instruments assess the reporting quality and internal and external validity simultaneously even though the consequences associated with poor reporting, the risk of bias, or the generalizability of the results are different [39]. Therefore, two tools were used to assess the quality of each study using the CAMARADES checklist. Overall, the median quality score for the 12 included studies was poor (5.29; interquartile range: 5–6), with scores ranging from 4 to 8. No study received a score of 0 or 10. Three studies received scores that indicated high quality [27, 28, 33], and these 3 studies reported the monitoring of physiological parameters during surgical procedures. One study [34] did not report the randomization of animals into treatment groups. Three studies [31, 32, 35, 36] assessed dose–response relationships. Three studies [30, 31, 33] stated no potential conflict of interests. Only one study [28] contained a statement that outcome measures were assessed by experimenters who were blind to the treatment condition. Moreover, no study described the sample size calculation to confirm that sufficient power had been achieved. The median quality score indicated that 17.5 out of 21 of the secondary criteria had been reported. The lowest score was 15 items (16.67 %) and the highest score was 19 items (8.33 %). Although treatment blinding and a description of the number of animals that died or were otherwise removed from the study are key measures for assessing the quality of studies, no study reported this information. One study [34] reported that investigators were blind to the treatment condition during the outcome assessment, and none of the papers described randomizing the order of the outcome assessments across the groups.

### Overall efficacy

**Fig. 2:**
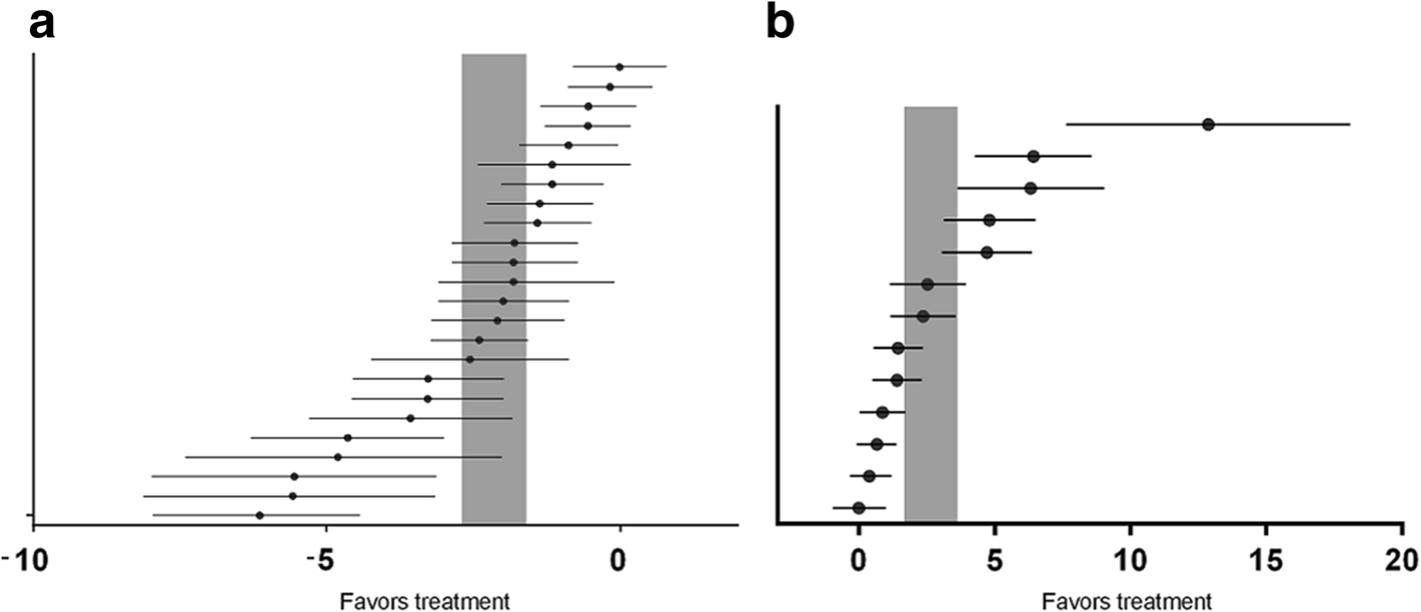
The effects of ginsenosides on (**a**) acquisition memory and (**b**) retention memory. The horizontal lines represent the mean estimated effect size and the 95 % confidence interval (CI) for each comparison. The vertical gray bars represent the 95 % CI of the pooled estimate effect size

For acquisition memory, the global estimated effect of ginsenosides was −2.14 (95 % CI: −2.69 to −1.79, *p* < 0.0001) with significant heterogeneity among studies (*X*^2^ = 136.74, df = 30, *p* < 0.0001, *I*^2^ = 83 %; Fig. 2a). For retention memory, the global estimated effect of ginsenosides was 2.65 (95 % CI: 1.67 to 3.64, *p* < 0.0001), with significant heterogeneity among studies (*X*^2^ = 108.89, df = 12, *p* < 0.0001, *I*^2^ = 89 %; Fig. 2b).

### Stratified meta-analysis

Subgroup analyses were conducted to assess the degree to which methodological differences between trials might have systematically influenced the differences observed in the primary treatment outcomes. The overall summary of each subgroup then can be inspected for evidence of variation in the effects of the intervention, which would suggest that the stratifying characteristic is an important source of heterogeneity and may modify the treatment efficacy. Current guidelines recommend at least 10 studies per characteristic for stratifying subgroups [40]. The results of the stratified analyses are described in Table 5.

**Table 5:** The results of stratified meta-analysis

Subgroups	Acquisition memory				Retention memory			
	Studies	Participants	Effect size, 95 % CI		Studies	Participants	Effect size , 95 % CI	
Gensenosides								
Rb	2	56	−2.21 [−2.90, −1.53]	*P* < 0.00001				
Rg1	13	290	−2.86 [−3.83, −1.90]		9	200	3.52 [1.95, 5.09]	*P* = 0.01
Rg2	3	72	−1.31 [−1.82, −0.79]		4	88	1.40 [−0.84, 1.97]	
Rg3	3	36	−2.56 [−4.42, −0.71]					
Rg5	3	72	−0.46 [−0.96, 0.04]					
Dose								
1–9 mg	9	216	−1.287 [−1.92, −0.62]	*P* = 0.009	7	172	1.17 [0.42, 1.93]	*P* = 0.002
10 mg	9	206	−2.54 [−3.49, −1.59]		3	60	6.54 [1.97, 11.1]	
20 mg	5	92	−2.90 [−4.51, −1.28]		3	56	4.30 [2.22, 6.37]	
30 mg	1	12	−4.81 [−7.41, 2.20]					
Animal species								
Mouse	6	136	−1.21 [−1.87, −0.55]	*P* = 0.008	4	96	1.35 [0.34,2.35]	*P* = 0.002
Rat	18	390	−2.52 [−3.24, −1.80]		9	192	3.52 [2.02,5.01]	
Gender								
Male	11	258	−2.65 [−3.66, −1.63]	*P* = 0.0008	5	256	3.22 [1.66, 6.26]	*P* = 0.003
Female	5	84	−3.42 [−4.84, −2.00]		5	84	3.78 [1.38, 6.10]	
Mixed	3	72	−1.31 [−1.82, −0.79]		3	73	1.22 [0.70, 1.73]	
Unclear	5	112	−0.86 [−1.46, −0.26]					
Anesthetic agent								
Pentobarbital	4	52	−2.22 [−3.40, −1.05]	*P* = 0.06	1	16	2.53 [1.13, 3.94]	*P* = 0.87
Chloral hydrate	14	322	−2.66 [−3.52, −1.80]		12	272	2.68 [1.63, 3.72]	
Ether	2	40	−1.58 [−2.32, −0.85]					
Unclear	4	112	−0.95 [−1.96, 0.06]					
Drug delivery								
ip	17	366	−2.23 [−2.87, −1.58]	*P* = 0.68	9	200	1.61 [0.73, 2.49]	*P* = 0.002
0r	7	160	−1.95 [−3.09, −0.81]		4	88	4.50 [2.92, 6.08]	
Study quality								
4	2	50	−4.05 [−8.02, −0.08]	*P* < 0.00001	1	20	2.36 [1.16, 3.56]	*P* < 0.001
5	3	72	−1.31 [−1.82, −0.79]		6	132	1.22 [2.92, 6.08]	
6	11	260	−1.90 [−2.63, −1.17]		4	88	4.50 [2.92, 6.08]	
7	2	60	−0.36 [−0.87, 0.15]		6	124	0.52 [0.00, 1.03]	
8	6	84	−3.66 [−5.20, −2.13]		3	48	5.98 [−0.63, 12.59]	

**Fig. 3:**
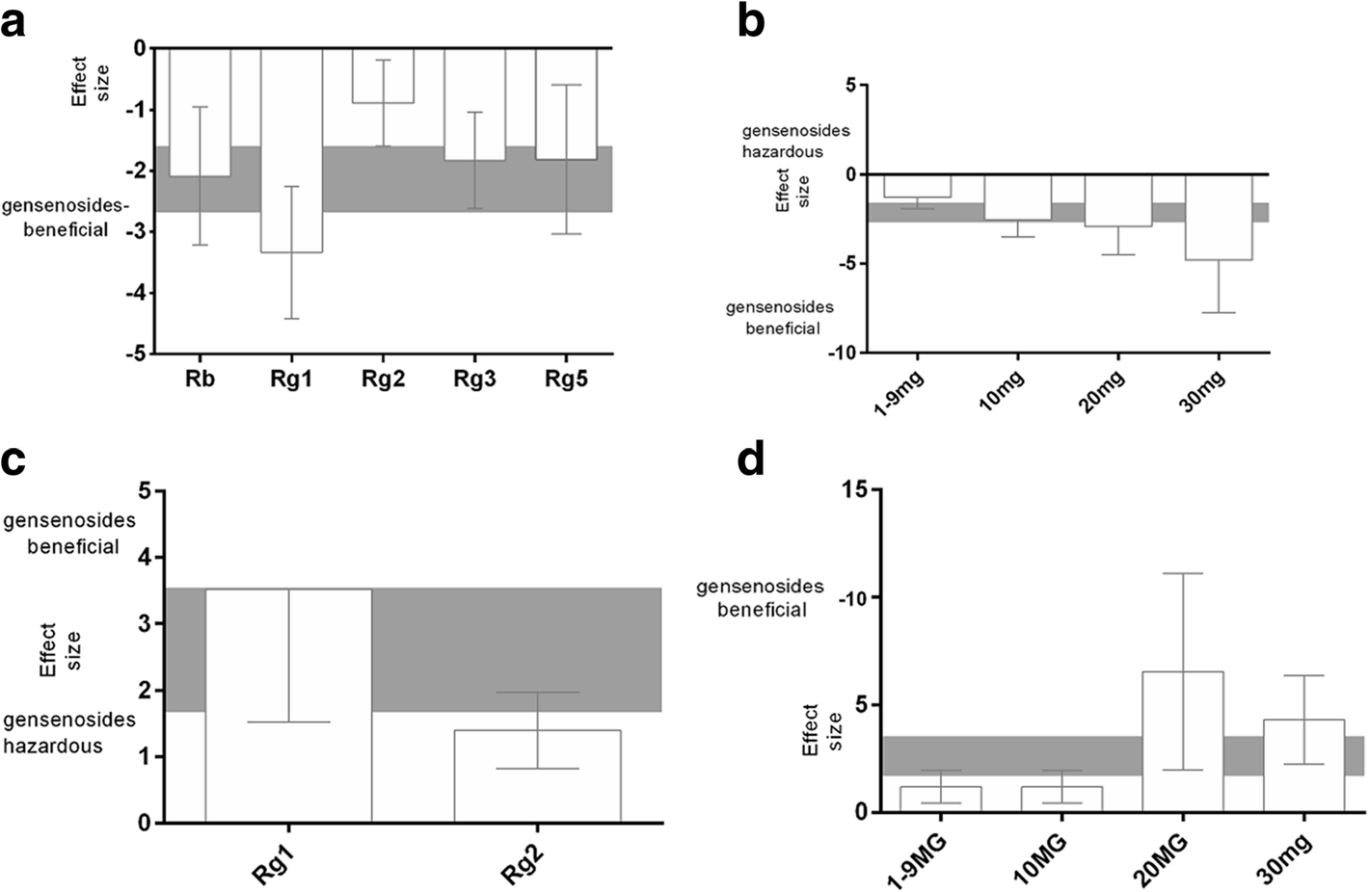
The effect size for acquisition memory is stratified by (**a**) the type of Ginsenosides; and (**b**) the dose. The effect size for retention memory is stratified by (**c**) the type of Ginsenosides; and (**d**) the dose. The gray bands represent the 95 % CI for the global estimated effect size

First, the protective effects of ginsenoside Rb, ginsenoside Rg1, ginsenoside Rg2, ginsenoside Rg3, and ginsenoside Rg5 administration on cognitive performance were examined. Rg1 treatment had a significantly greater beneficial effect on acquisition memory and retention memory (*X*^*2*^ = 29.55, df = 4, *p* < 0.00001, *I*^*2*^ = 86.5 % and *X*^*2*^ = 6.15, df = 1, *p* = 0.001, *I*^*2*^ = 83.7 %, respectively) compared with Rb treatment, Rg2 treatment, Rg3 treatment, or Rg5 treatment. Next, the efficacy of different doses of ginsenosides on cognitive performance was analyzed. For both acquisition and retention memory, significant beneficial effects were noted for all doses of ginsenosides. The protective effects of 30 mg or higher doses on acquisition memory were examined to determine whether the effects of higher doses are greater than those of lower doses. A 30 mg or higher dose was associated with a greater beneficial effect than a dose less than 30 mg on acquisition memory; however, no significant differences among doses were detected (*X*^*2*^ = 11.64, df = 3, *p* = 0.009, *I*^*2*^ = 74.2 %). The protective effects at 10 mg or higher doses on retention memory also were examined to determine whether the effects of higher doses are greater than lower doses, and a significant effect was found. A 10 mg dose was associated with a significantly greater beneficial outcome compared with a dose less than 10 mg or more than 10 mg for retention memory (*X*^*2*^ = 12.16, df = 2, *p* < 0.002, *I*^*2*^ *=* 83.5 %; Fig. 3).

**Fig. 4:**
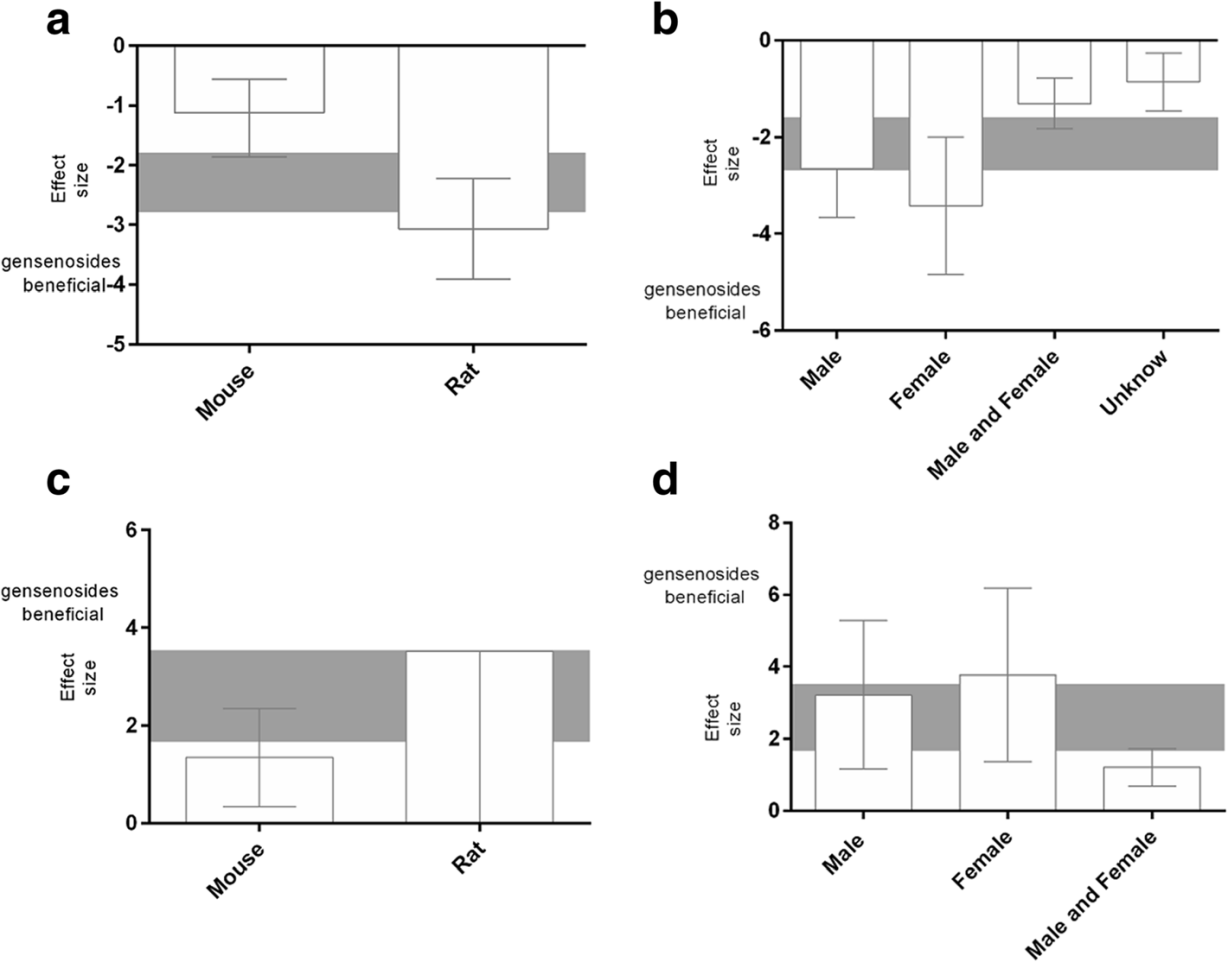
The effect size for acquisition memory is stratified by (**a**) species; and (**b**) sex. The effect size for retention memory is stratified by (**c**) species; and (**d**) sex. The gray bands represent the 95 % CI for the global estimated effect size

The protective effects of species and sex also were examined. For acquisition memory, although the effect size was higher in studies that used rat models, no significant difference was detected between studies that used rat models or mouse models (*X*^*2*^ = 6.97, df = 1, *p* = 0.008, *I*^*2*^ = 85.7 %). However, for retention memory, the effect size was significantly higher in studies that used rat models compared with mouse models (*X*^*2*^ = 5.58, df = 1, *p* = 0.002, *I*^*2*^ = 82.1 %). For both acquisition memory and retention memory, the effect size was significantly higher in studies that used female animals compared with male animals (*X*^*2*^ = 16.72, df = 3, *p* < 0.0008, *I*^*2*^ = 82.5 % and *X*^*2*^ = 7.25, df = 1, *p* = 0.003, *I*^*2*^ = 72.4 %, respectively; Fig. 4).

**Fig. 5:**
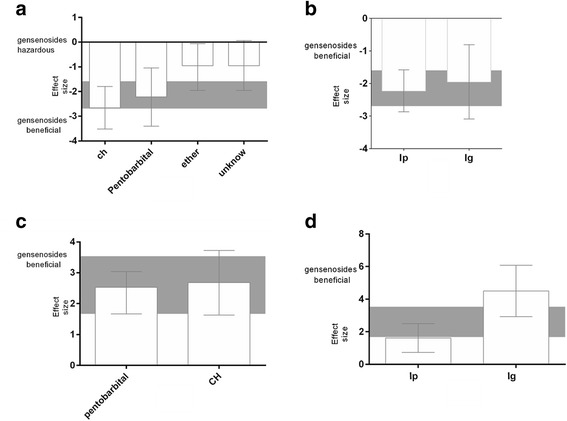
The effect size for acquisition memory is stratified by (**a**) anesthetic agent; and (**b**) route of delivery. The effect size for retention memory is stratified by (**c**) anesthetic agent; and (**d**) route of delivery. The gray bands represent the 95 % CI for the global estimated effect size

The effect of the anesthetic agent and the route of drug delivery also were examined. For acquisition memory and retention memory, although the effect size was higher in studies that used chloral hydrate anesthesia, no significant differences among anesthetic agents were detected (*X*^*2*^ = 7.38, df = 3, *p* = 0.006, *I*^*2*^ = 53.6 % and *X*^*2*^ = 0.03, df = 1, *p* = 0.87, *I*^*2*^ = 0 %, respectively; Fig. 5). For acquisition memory, although the effect size was higher in studies that used intraperitoneal injection, no significant differences in the routes of drug delivery were detected (*X*^*2*^ = 0.17, df = 1, *p* = 0.68, *I*^*2*^ = 0 %; Fig. 5c). Oral gavage was associated with a significantly greater beneficial outcome than intraperitoneal injection for retention memory (*X*^*2*^ = 9.83, df = 1, *p* = 0.002, and *I*^*2*^ = 89.8 %; Fig. 5d).

**Fig. 6:**
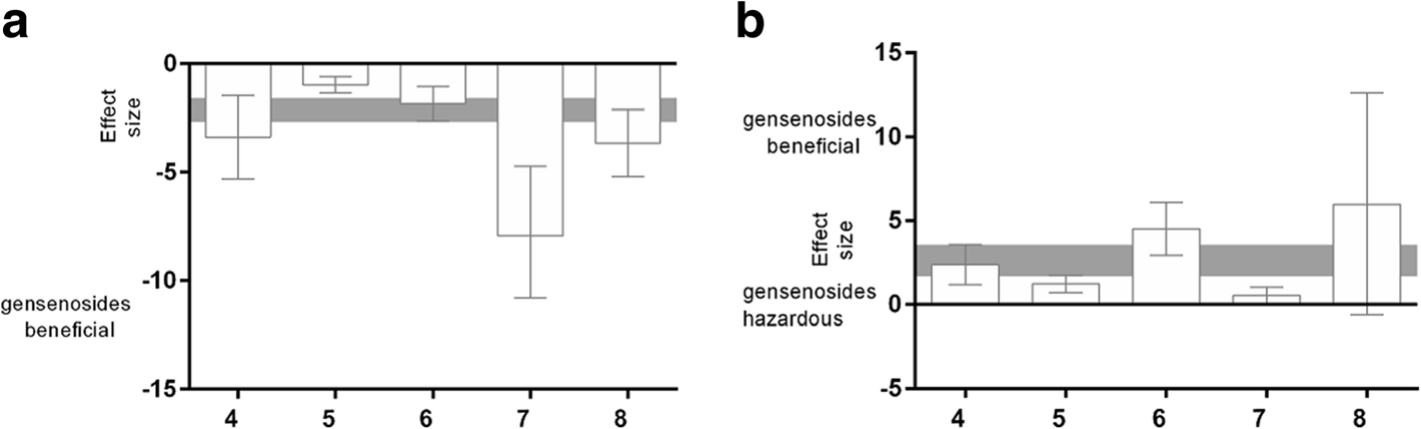
The effect size is stratified by study quality score (**a**) for acquisition memory and (**b**) for retention memory. The gray bands represent the 95 % CI for the global estimated effect size. The gray bands represent the 95 % CI for the global estimated effect size

The effect sizes for acquisition and retention memory also were examined relative to the study quality score. Significant differences in effect sizes were observed between lower-scoring and higher-scoring studies for both acquisition (*X*^*2*^ = 25.03, df = 4, *p* < 0.00001, *I*^*2*^ = 84.3 %) and retention (*X*^*2*^ = 29.03, df = 4, *p* < 0.00001, *I*^*2*^ = 86.2 %) memory. The effect size for acquisition memory was maximal for studies with a quality score of 4 (−4.05, 95 % CI: −8.02 to −0.08; Fig. 6a), and the effect size for retention memory was higher for studies with a quality score of 8 (5.98, 95 % CI: −0.63 to 12.59; Fig. 6b) than those with lower scores.

### Meta-regression analyses

Meta-regression is an extension of the subgroup analysis that allows for the investigation of the effect of multiple factors simultaneously. The outcome variable is the effect estimate, and the explanatory variables are the study characteristics that might influence the effect size, which are often called the “potential effect modifiers” or covariates.

To further explore the heterogeneity among studies, meta-regression was conducted for the acquisition and retention memory results. For retention memory, the species and sex of the animals, anesthetic method, type and dose of ginsenoside, study quality score, and route of drug delivery explained 49.03 % of the heterogeneity. For acquisition memory, heterogeneity was independent of these factors.

### Publication bias

**Fig. 7:**
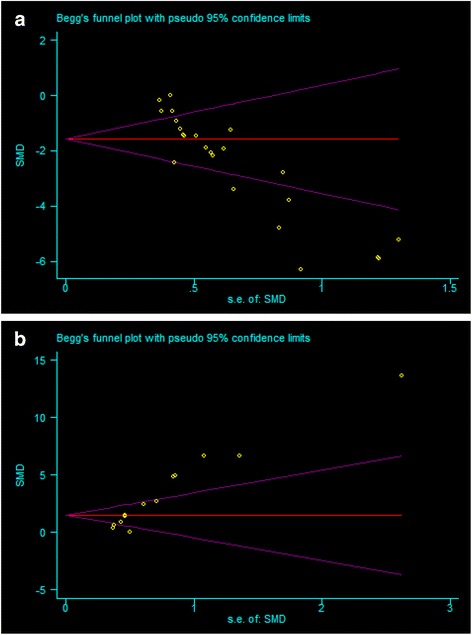
Funnel plot for acquisition memory (**a**) and retention memory (**b**)

Finally, the presence of small-study effects, which may contribute to publication bias, were identified. Funnel plots showed an asymmetry for both the acquisition (Fig. 7a) and retention memory (Fig. 7b) data, which provides evidence for small-study effects (Egger regression, *p* < 0.0001 and *p* < 0.001, respectively).

### Possible drug protection mechanism analysis

All studies that were included in the analysis assessed the biological mechanisms of ginsenoside activity. Across studies, the neuroprotective effect of ginsenoside was attributed primarily to anti-inflammatory activity [28, 32, 34]. Ginsenoside was reported to promote the non-amyloidgenic cleavage of beta-amyloid precursor protein (APP) [18], attenuate Aβ formation [29], decrease Aβ levels, attenuate hippocampal histopathological abnormalities [30], prevent tau hyperphosphorylation via the regulation of p-GSK3 and serine/threonine-specific protein phosphatase 2A levels [31], activate the endoplasmic reticulum signaling pathway, inhibit the activity of acetylcholinesterase [37], and upregulate the expression of nerve growth factor [36].

## Discussion

Many animal experiments are performed to inform human health, and may play an important role in the identification and development of drugs, medical devices, and surgical procedures; risk assessments for safe human exposures; and increasing biological knowledge. It would seem rational to critically review the existing relevant animal experiments before new animal experiments and, in particular, clinical trials in humans are performed. Systematic reviews and meta-analyses are suitable tools to summarize the current evidence on a given subject, and therefore directly support the ‘three Rs’ (i.e., replacement, reduction, and refinement) by, for example, preventing the unnecessary duplication of animal studies. Systematic reviews and meta-analyses play an important role in physics, the social sciences, and medicine [41].

The results of this systematic review and meta-analysis show that ginsenoside provides neuroprotective effects in terms of improving cognitive outcomes in AD. Ginsenoside Rg1 exhibited the highest protective effect on both acquisition and retention memory. The species and sex of the animals, the type and dose of ginsenoside, and the study quality all had significant impacts on the effect size. In contrast, the route of drug delivery and the anesthetic method had no significant effects on the outcomes. Our analysis also suggests that some aspects of the original study design had an impact on the study outcome. First, the effect size was higher in rat studies than in mouse studies, which suggests that different species may react differently to ginsenoside. Second, the route of administration and dosage of ginsenoside also affected the outcome [42]. The protective effect on acquisition memory was better with doses of 30 mg or higher (although not significantly) and the protective effect on retention memory was better with doses of 10 mg. These results are not consistent with the dose-linear response curve described previously, in which higher doses yielded a greater response [43]. The effect size probably was overstated in studies that administered lower doses.

The methodological quality of the studies was assessed according to standards previously described for the preclinical development of neuroprotective drugs with minor modifications [22, 24]. In general, the quality of the included studies was poor. For example, only one study reported that investigators were blind to the treatment condition during the behavioral assessments. Treatment blinding is recommended in open-label trials to reduce bias. If patients, clinicians, or assessors are aware of the treatment assignment, then this knowledge may influence the reporting or measurement of the outcome and introduce bias [44]. Moreover, all the studies failed to report the calculation of the sample size necessary to achieve sufficient power, which is crucial to judge the efficacy of a new therapy or drug [45]. Unfortunately, the reporting of sample size elements specific to these random trials remains below that necessary for transparent reporting. The authors should calculate the sample size during the planning phase of the study to assess the accuracy of the a priori estimates and aid the design of future trials. In addition, journal editors and peer reviewers should implement stricter requirements for authors to follow CONSORT recommendations [46]. All studies failed to report animals that died or were otherwise removed from the study. As these events may have been due to medication side effects, this information is important in assessing the use of ginsenoside.

Systematic reviews can use research data from numerous study designs. However, when conducting systematic reviews of interventions, studies conducted using low evidentiary designs for evaluating real-world efficacy are generally not appropriate for inclusion [47]. In our systematic review, high quality studies showed a trend toward better acquisition memory outcomes, but studies with lower quality scores exhibited the highest protective effect on retention memory. The effect size was probably overstated in studies with lower quality scores.

Our study has several limitations. First, although we conducted a thorough literature search, we did not conduct a search of older data that has not been indexed in an electronic/online database. Our analysis is based only on published data or academic dissertation data available online, most of which showed positive results; therefore, our study may have missed ‘negative’ results. In addition, positive results, which are easier to publish, often appear in journals with higher impact factors than negative results [48]. The funnel plots and Egger tests suggest the possibility of a publication bias or other small-study bias, which is consistent with observations from other systematic reviews of animal studies [49]. Publication bias, which is considered a potential threat to the validity of all systematic reviews that include experimental studies, may have led to an overestimation of the protective effect of ginsenoside in our study. Second, we focused only on the effect of ginsenoside on cognitive deficits in AD. We did not conduct analyses to investigate the effect on histopathology, such as plaques and tangles, due to insufficient data. Third, due to language barriers, we only searched databases for articles published in English or Chinese, and did not search for studies published in other languages such as Korean. Korea is one of the main countries that uses ginseng, and therefore we may have missed some relevant publications. In addition, the animals used in the included studies were young rodents, which is not consistent with the average age of humans in the relevant clinical setting of AD. There remain some unknown factors that contributed to the heterogeneity of the effect size in our study. The number of preclinical experiments performed each year continues to increase, and our understanding of the disease mechanism is improving. However, the number of novel interventions that reach the clinic to treat cerebrovascular diseases continues to fall due to limitations in the translational paradigm [50]. The standardization of animal protocols and the systematic review of animal models that do not currently qualify as predictive modalities for human responses to drugs and disease are supported by experts in various fields of science [51]. Therefore, these limited results may not be adequate for the transition from animal experiments to human clinical trials. Consequently, prior to making any clinical practice recommendations, high methodological reporting and quality control experimental studies are needed to better evaluate the impact of this promising pharmacological intervention for AD.

## Conclusion

This systematic review and meta-analysis indicates that treatment with ginsenoside can alleviate cognitive deficits in experimental animal models of AD. Although some factors, such as the study quality and a potential publication bias, may undermine the validity of these positive findings, ginsenosides may play a potential neuroprotective role in AD. However, without rigorous, robust, and detailed preclinical evaluations, novel neuroprotective drugs may prove to be ineffective when tested in large, time-consuming, and expensive human clinical trials. Therefore, additional well-designed and well-reported experimental animal studies are needed.
